# Structural basis of human LRG1 recognition by Magacizumab, a humanized monoclonal antibody with therapeutic potential

**DOI:** 10.1107/S2059798322004132

**Published:** 2022-05-09

**Authors:** Javier Gutiérrez-Fernández, Faiza Javaid, Giulia De Rossi, Vijay Chudasama, John Greenwood, Stephen E. Moss, Hartmut Luecke

**Affiliations:** a Structural Biology and Drug Discovery Group, Centre for Molecular Medicine Norway, Nordic EMBL Partnership, University of Oslo and Oslo University Hospital, 0318 Oslo, Norway; b Institute of Ophthalmology, University College London, London, United Kingdom; c Department of Chemistry, University College London, London, United Kingdom; d Department of Physiology and Biophysics, University of California, Irvine, Irvine, CA 92697, USA

**Keywords:** cancer immunotherapy, leucine-rich α-2-glycoprotein 1, Magacizumab, angiogenesis, antibody–antigen complex

## Abstract

The formation of new dysfunctional blood vessels is a crucial stage in the development of various conditions such as macular degeneration, diabetes, cardiovascular disease, neurological disease and inflammatory disorders, as well as during tumor growth, eventually contributing to metastasis. An important factor involved in pathogenic angiogenesis is leucine-rich α-2-glycoprotein 1 (LRG1), the antibody blockade of which has been shown to lead to a reduction in both choroidal neovascularization and tumor growth in mouse models. In this work, the structural interactions between the LRG1 epitope and the Fab fragment of Magacizumab, a humanized function-blocking IgG4 against LRG1, are analysed, determining its specific binding mode and the key residues involved in LRG1 recognition. Based on these structural findings, a series of mutations are suggested that could be introduced into Magacizumab to increase its affinity for LRG1, as well as a model of the entire Fab–LRG1 complex that could enlighten new strategies to enhance affinity, consequently leading towards an even more efficient therapeutic.

## Abstract 2

Structural interactions between the LRG1 epitope and the Fab fragment of Magacizumab determine its specific binding mode and the key residues involved in LRG1 recognition.

## 1. Introduction

Neoangiogenesis is a phenomenon that plays an important role in many diseases, including those affecting the eyes, kidneys and lungs, as well as promoting cancer growth. Vascular dysfunction, whether it arises from a failure of new vessels to stabilize or from existing vessels losing normal function, is driven by various factors that regulate the growth and functional stability of blood vessels (Wang *et al.*, 2013 ▸; Carmeliet & Jain, 2000 ▸; Potente *et al.*, 2011 ▸). While vascular endothelial growth factor (VEGF) is a major player in this context, other secondary factors also contribute to vascular signaling in disease. Such is the case for TGF-β1, TGF-β type II receptor, ALK1, Smad1, Smad5 and Smad8, the regulation of which depends on multiple factors and the presence of additional modulatory proteins. In summary, these pathways involve the interaction of many factors, some as yet unknown, complicating the development of therapies and treatment strategies targeting the previously mentioned diseases.

By studying pathological retinal vascularization in a range of mouse mutants, Wang *et al.* (2013 ▸) found leucine-rich α-2-glycoprotein 1 (LRG1; a glycosylated 50 kDa protein with 347 residues in humans) to be induced nearly exclusively in this vasculature. LRG1 belongs to the leucine-rich repeat family of proteins, many of which are involved in protein–protein interactions and cell-signaling pathways. Further analysis determined that under normal conditions LRG1 is not present in the systemic vasculature and is not involved in developmental angiogenesis, only being present to any extent in the liver and in neutrophils. Thus, the normal function of LRG1 has yet to be elucidated, although there is evidence that it may be involved in wound healing (Gao *et al.*, 2020 ▸; Pickert *et al.*, 2009 ▸) and also in protection against the pro-apoptotic effects of extracellular cytochrome *c* (Shirai *et al.*, 2009 ▸; Jemmerson, 2021 ▸).

When studying disordered vascularization in mice following laser-induced choroidal neovascularization (CNV) and oxygen-induced retinopathy, Wang *et al.* (2013 ▸) further revealed that LRG1 gene deletion or blockade with an anti-LRG1 antibody resulted in a significant reduction in lesion size. These experiments, together with recent reports that LRG1 is involved in tumor growth, tumor metastasis and emphysema (Hisata *et al.*, 2021 ▸; Singhal *et al.*, 2021 ▸; O’Connor *et al.*, 2021 ▸), indicate that LRG1 affects vascular stability and may be a suitable therapeutic target in multiple pathologies associated with aberrant vascularization and vascular destabilization. Accordingly, a program was undertaken to develop a therapeutic antibody that was initiated with the generation of a cohort of more than 100 mouse monoclonal antibodies (mAbs) that specifically bind to human LRG1. The panel of mAbs was derived using conventional hybridoma technology following the immunization of mice with recombinant human LRG1. The entire cohort of mAbs was assayed for therapeutic suitability and reduced to three lead candidates based on their anti-angiogenic activity and affinity, with the anti-angiogenic activity providing a measure of the function-blocking activity of each mAb. Among these three was 15C4 which, due to its specificity and near-irreversible picomolar affinity, was humanized with a view to future testing in clinical trials (Kallenberg *et al.*, 2020 ▸). Humanized antibodies generally comprise an Fc region from IgG1, IgG2, IgG3 or IgG4 and a Fab region containing the paratope. The paratope, as well as its surroundings, is crucial for specificity against the target and may be engineered to increase the affinity of the antibody–antigen complex. In the case of 15C4, humanization resulted in a loss of affinity of approximately an order of magnitude, suggesting that affinity maturation may be both desirable and feasible. For the same purpose, obtaining structural information on the binding mechanism between the components will help not only to understand complex formation but also to design new antibodies with improved affinity and/or specificity.

In this work, we have determined the high-resolution crystal structure of the Fab fragment of Magacizumab (Maga_Fab_), the lead IgG4 humanized from 15C4, in complex with a 15-amino-acid peptide that corresponds to the epitope in LRG1 (Kallenberg *et al.*, 2020 ▸). Careful analysis of the atomic interactions allowed us to identify LRG1 residues that play a key role in antibody recognition as well as the surrounding interactions that contribute to binding and strengthen Maga_Fab_–LRG1 complex formation. Lastly, to extract insights into the overall organization of the complex between full-length LRG1 and Maga_Fab_, a homology model of LRG1 was built and structurally aligned with the peptide posed over the paratope.

## 2. Materials and methods

### 2.1. Antibody generation, Fab cleavage and Fab–peptide purification

Magacizumab was generated as a recombinant antibody using a CHO cell-based expression system and purified from the culture medium as described previously (Kallenberg *et al.*, 2020 ▸). The antibody was formulated at 75.4 mg ml^−1^ in digestion buffer (20 m*M* monobasic sodium phosphate, 10 m*M* disodium EDTA, 80 m*M* cysteine–HCl pH 7.2) and reacted with a 1:10(*w*:*w*) amount of immobilized papain (Thermo Scientific; 250 µg per milligram of gel) by incubation for 24 h at 37°C whilst shaking (at 1100 rev min^−1^). Cysteine–HCl was added immediately before Magacizumab digestion. After digestion, the resin was separated from the digest using a filter column and washed with phosphate-buffered saline (PBS) pH 7.0 three times. The digest was combined with the washes, the buffer was exchanged completely for PBS pH 7.4 using diafiltration columns (10 kDa molecular-weight cutoff) and the volume was adjusted to 2 ml. The sample was then applied onto an NAb protein A column (Thermo Scientific). The Fab fraction, Magacizumab Fab (Maga_Fab_), was eluted according to the manufacturer’s protocol; the column was washed three times with PBS pH 7.4 and the Fc fraction was eluted with 0.2 *M* glycine–HCl pH 2.5, which was neutralized with 10% of the volume of 1 *M* Tris pH 8.5 solution. The Maga_Fab_ fraction was combined with the washes and both the Fab and Fc solutions were buffer-exchanged into PBS pH 7.4 using diafiltration columns (10 kDa molecular-weight cutoff). The digests were analyzed by SDS–PAGE and LCMS to reveal the formation of Maga_Fab_. The observed molecular mass was 47 461 Da, while the computed mass was 47 412 Da.

### 2.2. Crystallization and structure determination of apo Maga_Fab_

Rod-shaped Maga_Fab_ crystals of approximately 100 × 50 × 50 µm in size were obtained by sitting-drop crystallization (0.5 µl sample and 0.5 µl well condition) at 23°C using the JCSG screen (Qiagen, Germany). Further optimization of the hit conditions led to larger needle-shaped crystals that grew in 0.2 *M* NaCl, 0.1 *M* bis-Tris pH 5.5, 25%(*w*/*v*) PEG 3350 at a protein concentration of 5 mg ml^−1^. The best crystals were cryoprotected using 0.1 *M* bis-Tris pH 5.5, 35%(*w*/*v*) PEG 3350 (Teng & Moffat, 2000 ▸). X-ray diffraction data were collected from several crystals at 100 K on the XALOC beamline at ALBA, Spain (Juanhuix *et al.*, 2014 ▸). Diffraction images were processed with *XDS* (Kabsch, 2010 ▸) and *AIMLESS* (Evans, 2011 ▸; Winn *et al.*, 2011 ▸), resulting in a 2.7 Å resolution *P*2_1_ data set expected to contain two molecules per asymmetric unit (36.5% solvent content). Molecular replacement was performed with *Phaser* (McCoy *et al.*, 2007 ▸) using the crystal structures of Pembrolizumab (PDB entry 5dk3, CH and CL chains; Scapin *et al.*, 2015 ▸), antibody B43 (PDB entry 6al4, VH chain; Teplyakov *et al.*, 2018 ▸) and humanized 5c8 Fab (PDB entry 6bjz, VL chain; Henderson *et al.*, 2020 ▸) as the highest homology templates for the respective chains. Several rounds of rebuilding and refinement were performed with *Coot* (Emsley *et al.*, 2010 ▸) and *Phenix* (Liebschner *et al.*, 2019 ▸). This apo Maga_Fab_ structure was not deposited in the PDB but was used as a molecular-replacement model to determine the structure of Maga_Fab_ in complex with the LRG1 epitope peptide.

### 2.3. Crystallization and structure determination of Maga_Fab_ in complex with the epitope peptide

The Maga_Fab_–peptide complex was obtained by co-crystallization at Maga_Fab_:peptide molar ratios of 1:2 and 1:5 using a Maga_Fab_ concentration of 12 mg ml^−1^ in 0.2 *M* NaCl, 0.1 *M* bis-Tris pH 5.5, 25%(*w*/*v*) PEG 3350. The best crystals were cryoprotected using 0.1 *M* bis-Tris pH 5.5, 35%(*w*/*v*) PEG 3350 (Teng & Moffat, 2000 ▸). X-ray diffraction data were collected from several crystals at 100 K on the XALOC beamline at ALBA, Spain (Juanhuix *et al.*, 2014 ▸). Diffraction images were processed with *XDS* (Kabsch, 2010 ▸) and *AIMLESS* (Evans, 2011 ▸; Winn *et al.*, 2011 ▸), resulting in a 1.7 Å resolution *P*2_1_2_1_2_1_ data set that was expected to contain two Maga_Fab_ molecules per asymmetric unit (56.9% solvent content; Table 1 ▸). Molecular replacement was performed with *Phaser* (McCoy *et al.*, 2007 ▸) using our previously determined apo Maga_Fab_ structure as a template. Several rounds of rebuilding and refinement were performed with *Coot* (Emsley *et al.*, 2010 ▸) and *Phenix* (Liebschner *et al.*, 2019 ▸).

Maga_Fab_ 1 (chains *A* and *B*), most of Maga_Fab_ 2 (chains *C* and *D*) and both peptide molecules (chains *E* and *F*) fit very well into the electron density (Supplementary Fig. S1), showing excellent geometry and no outliers (as assessed by *MolProbity*; Chen *et al.*, 2010 ▸). However, a region of about 60 residues located after Ser25 of chain *D* (corresponding to the VH chain of Maga_Fab_ 2) displayed significant *F*_o_ − *F*_c_ positive density at contouring levels below 5σ along the main chain and side chains. In general, this positive difference density displays the overall features and shape of several side chains shifted between 2 and 3 Å in defined directions, as well as a thickening of the main chain. The magnitude of these features varies in range depending on the area, consistent with a pseudo-rigid-body displacement and slight torsion of the whole region (Supplementary Fig. S2*a* ). Different molecular-replacement and refinement strategies were performed to minimize this positive difference density. Being unable to obtain electron-density maps that lacked the positive density in this region with a single conformation for chain *D*, we explored the possibility of differential crystal contacts and a global double conformation affecting not only isolated residues but a substantial area of the VH region of molecule 2 (chain *D*). The best structure, with an *R* factor of 0.182 and an *R*_free_ of 0.206 (Table 1 ▸), was obtained by building two fragments in a double conformation (Ser25–Val37 and Ile48–Ser85) and by refining *XYZ* in both reciprocal and real space, translation–libration–screw rotation (TLS) factors, occupancies and individual *B* factors without applying noncrystallographic symmetry (NCS) restraints (Usón *et al.*, 1999 ▸; Supplementary Figs. S2*b* and S2*c* ). The final occupancies were 0.47:0.53 for the first fragment and 0.43:0.57 for the second fragment. Although scattered *F*_o_ − *F*_c_ positive density was still visible in some minor exposed areas, building a double conformation of these two regions decreased both *R* factors by about 2% compared with the same strategy performed with a single conformation at an occupancy of 1.00 as a control. Composite omit maps and refinements of partial models were performed to minimize bias during the whole process.

In summary, Maga_Fab_ 1 (chains *A* and *B*), as well as its respective peptide (chain *E*), do not suffer from differential crystal contacts, hence the single conformation combined with the high quality of both the maps and model in these regions allowed us to describe and analyze both the overall Maga_Fab_ structure and its interactions with the LRG1 epitope at the reliability expected for a 1.7 Å resolution study.

### 2.4. Three-dimensional homology modeling

Three-dimensional homology models of mouse hybridoma 15C4 variable domains were generated with *SWISS-MODEL* (Waterhouse *et al.*, 2018 ▸). The best model of the murine hybridoma 15C4 VH chain, according to the GMQE (global model quality estimation), was built using the pyroglutamate-amyloid-β-specific Fab c#24 heavy chain (PDB entry 5myx, chain *B*) as a template (Piechotta *et al.*, 2017 ▸), returning a GMQE of 0.97, a sequence identity of 83.9%, a sequence similarity of 90.7% and a coverage of 97.0%. The VL chain of the murine hybridoma 15C4 Fab was generated using the VL fragment of *Mus musculus* IgG as determined in the crystal structure of the anion-exchanger domain of human erythrocyte band 3 (PDB entry 4yzf, chain *F*; Arakawa *et al.*, 2015 ▸) as a template. In this case the GMQE was 0.99, with 89.2% sequence identity and 95.5% sequence similarity with 100% coverage.

A three-dimensional homology model of LRG1 was built with *SWISS-MODEL* using NetrinG ligand 2 (NGL2; PDB entry 3zyi, chain *A*) as a template (Seiradake *et al.*, 2011 ▸), returning a GMQE of 0.59, a sequence identity of 22.1%, a sequence similarity of 39.9% and a coverage of 86.0%. Additional models were built by threading with *RaptorX* (Peng & Xu, 2011 ▸; Xu *et al.*, 2021 ▸). Up to five models were predicted, with estimated Cα r.m.s.d.s ranging between 2.02 and 2.13 Å.

## 3. Results and discussion

### 3.1. Overall structure

**Figure 1:**
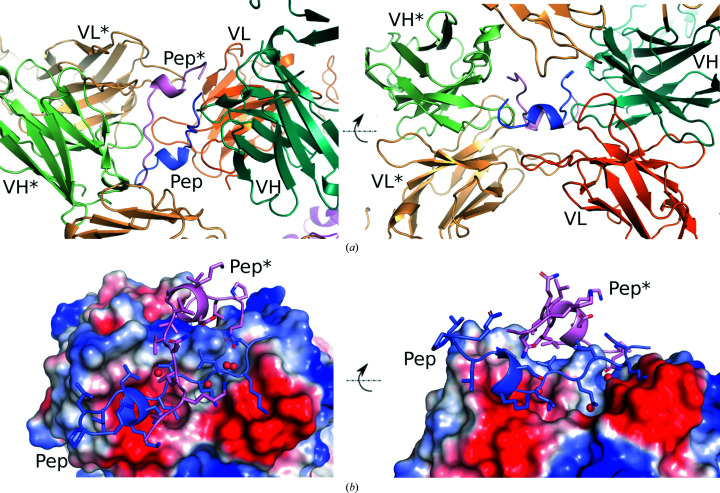
Crystal packing and peptide arrangement. (*a*) The asymmetric unit consists of two peptide molecules (labeled Pep and Pep*; purple and pink, respectively) packed between two Maga_Fab_ molecules (Maga_Fab_ molecule 1 light chains labeled VH/VL; Maga_Fab_ molecule 2 light chains labeled VH*/VL*). A third neighboring VL chain (orange, not labeled) from a different asymmetric unit establishes weak interactions with one of the peptides. (*b*) Electrostatic surface showing polar pockets for the peptide interaction and important structural waters. Red indicates a negatively charged surface, while blue indicates positive charge.

The crystal structure of Maga_Fab_ in complex with the LRG1 epitope shows the 15-residue peptide (Pep; GNKLQVLGKDLLLPQ) bound at its paratope along the VL and VH chains of Maga_Fab_, forming a partially folded structure consisting of a short 3_10_-helix (8-GKDL-11) flanked by loop regions without specific secondary structure. In addition to this arrangement, a symmetry-related peptide molecule (Pep*) as well as a symmetry-related Maga_Fab_ molecule are located near the main peptide, establishing additional contacts that strengthen crystal packing (Fig. 1 ▸, Supplementary Fig. S3 and Table 2 ▸).

**Figure 2:**
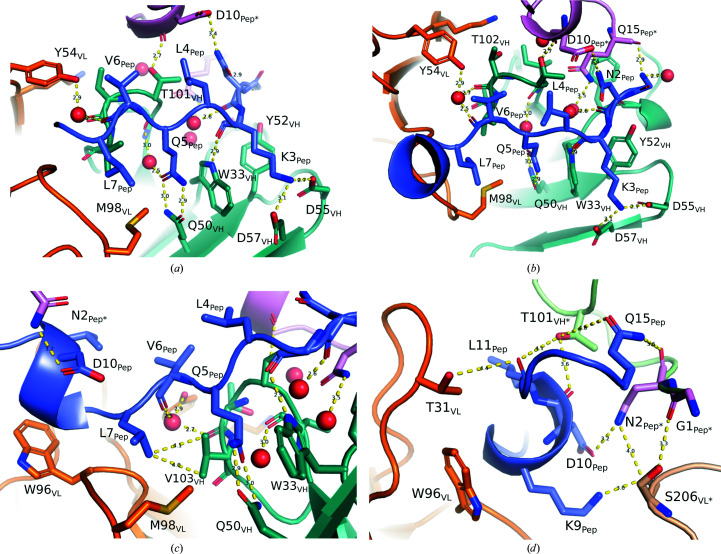
Main Maga_Fab_–peptide interactions. (*a*) The bidentate interaction between the amides of Gln5_Pep_ and Gln50_VH_, as well as the salt bridges between Lys3_Pep_ and Asp55_VH_ and Asp57_VH_, are the key interactions that determine the recognition of the peptide by the Fab. Additional weaker interactions and those involving structural waters are also represented by yellow dashed lines. The VH chain is shown in green, the VL chain in orange, the main peptide in purple and the symmetric peptide in pink. (*b*) The same as (*a*) but from a different orientation. (*c*) Stabilization of the nonpolar section of the peptide. The short 3_10_-helix of the peptide is flanked by Trp96_VL_, while Leu7_Pep_ is located near Val103_VH_. The carbonyl group of Val6_Pep_ is stabilized by a water located between the hydroxyl group of Ser95_VL_ and the Tyr54_VL_ and Thr102_VH_ carbonyl groups. An interaction with the symmetric peptide is established between Asp10_Pep_ and Asn2_Pep*_. (*d*) Stabilization of the peptide 3_10_-helix moiety by symmetry molecules. Lys9_Pep_, Asp10_Pep_, Leu11_Pep_ and Gln15_Pep_ are stabilized by Ser206_VL_, Gly1_Pep*_, Asn2_Pep*_ and Thr101_VH_ from symmetry molecules (represented in light orange, light pink and light green, respectively).

The strongest interaction between Maga_Fab_ and the peptide is a pair of hydrogen bonds between the respective side-chain amides of Gln5_Pep_ and Gln50_VH_, supported by a water-mediated hydrogen bond to the carbonyl group of Ile100_VH_ (Fig. 2 ▸ *a*). This arrangement is flanked on one side by Trp33_VH_, which, in addition to a possible stacking effect, is likely to stabilize the main chain of Lys3_Pep_. This lysine is of special importance due to its Coulombic interactions with Asp55_VH_ and Asp57_VH_. In addition to these key interactions, the N-terminal Gln1_Pep_ is located between Tyr52_VH_, the carbonyl group of Gly31_VH_, the C-terminal hydroxyl group of Gln15_Pep*_ and a structural water molecule, while Asn2_Pep_ interacts with Asp10_Pep*_ from the symmetry-related peptide, completing the stabilization of this region (Fig. 2 ▸ *b*).

While the binding of the Gly1_Pep_–Gln5_Pep_ portion of the peptide mainly relies on a strong network of polar interactions with residues from the VH chain and water molecules, the second portion (Val6_Pep_–Gln15_Pep_) mainly establishes hydrophobic interactions between Leu7_Pep_ and Val103_VH_, as well as stacking interactions of the short 3_10_-helix in Pep with Phe36_VL_ and Trp96_VL_ (Fig. 2 ▸ *c*). Another key structural water, located between Tyr54_VL_ and the carbonyl group of Thr102_VH_, is involved in the stabilization of the peptide by its proximity to the carbonyl group of Val6_Pep_. Finally, a network of interactions between Lys9-Asp10-Leu11/Gln15_Pep_ and the symmetry-related neighbors (Gly1_Pep*_, Asn2_Pep*_, Ser206_VL*_ and Thr101_VH*_) completes the interactions of this region (Fig. 2 ▸ *d*). While these interactions are the strongest, the tight packing of the different molecules in the crystals leads to a significant contact surface area between the main peptide and a symmetry-related molecule (352.0 Å^2^), similar to the area between the main peptide and each of the Maga_Fab_ chains (*A*_Pep–VH_ = 356.0 Å^2^, *A*_Pep–VL_ = 338.2 Å^2^; Table 2 ▸). The contact areas between the main peptide and the symmetry-related Maga_Fab_ chains are smaller (*A*_Pep–VH*_ = 187.1 Å^2^, *A*_Pep–VL*_ = 14.2 Å^2^), but definitely contribute to crystal packing.

### 3.2. Strategies for increasing the affinity of Magacizumab for LRG1

From a therapeutic point of view, increasing the affinity of Magacizumab for LRG1 would be expected to provide certain benefits, since this would permit a potential reduction in dosage and frequency of administration to patients. Affinity maturation of an anti-VEGF Fab led to the development of ranibizumab (Lucentis), a therapeutic widely used in eye disease that has approximately a tenfold higher affinity for VEGF than the parental Fab (Chen *et al.*, 1999 ▸). The crystal structure of Maga_Fab_ in complex with its LRG1 epitope described in this work has allowed us to analyze and explore potential mutations that could similarly lead to a significant increase in affinity. Some key features that can be extracted from this analysis are that Gln50_VH_ is probably the main residue involved in the recognition of the antigen by strongly interacting with Gln5_Pep_, while Asp55_VH_ and Asp57_VH_ are required to stabilize Lys3_Pep_. The remaining interactions are either nonpolar or are mediated by structural waters.

While mutations of the key residues (Gln50_VH_, Asp55_VH_ and Asp57_VH_) could cause negative effects in the paratope, leading to loss of affinity, mutations of residues showing weaker or water-mediated interactions could result in new and stronger direct contacts with the epitope, hence increasing the stability of the complex. Such is the case for Trp33_VH_. Located between the bidentate Gln50_VH_–Gln5_Pep_ interaction and the Lys3_Pep_ side chain, it forms a hydrogen bond to the carbonyl group of the lysine. However, two important structural waters (W46 and W297) are located next to this position. Mutation of Trp33_VH_ to an arginine could place the guanidino group in the same position as these waters, presumably increasing the affinity of the peptide main chain (Supplementary Fig. S4*a* ). However, due to the proximity to the bidentate Gln–Gln interaction, an arginine in this position could potentially destabilize or prevent this strong interaction due to steric hindrance, causing the opposite of the desired effect.

Another interesting candidate is Gly31_VH_ since it is located next to Gly1-Asn2_Pep_ and is exposed to the solvent. Therefore, a mutation of this residue is less prone to interfere with other key residues. A short polar residue such an aspartate or a serine might be sufficient to create an interaction with Asn2_Pep_ and would not alter the current main-chain geometry, therefore keeping the φ/ψ values in the favored region of the non­glycine Ramachandran plot (Supplementary Fig. S4*b* ).

On the nonpolar section of the peptide, mutation of Met98_VL_ to a leucine might strengthen the hydrophobic interactions between Leu7_Pep_ and Val103_VH_ (Supplementary Fig. S4*c* ). A similar effect might be obtained by mutating Thr31_VL_ to a leucine or an isoleucine, placing a nonpolar chain between Leu11_Pep_ and Leu12_Pep_ to potentiate hydrophobic interactions with one of these side chains (Supplementary Fig. S4*d* ). However, due to the exposed location of both Thr31_VL_ and the peptide 3_10_-helix segment, the current spatial arrangement (4.4 Å between the Thr31_VL_ hydroxyl and Leu11_Pep_ carbonyl groups) could differ in the Maga_Fab_–LRG1 complex, where the distances between these residues might differ significantly.

### 3.3. Modeling of murine hybridoma 15C4 variable domains

To explore the differences in affinity between the mouse hybridoma 15C4 mAb and Maga_Fab_, three-dimensional homology models of 15C4 mAb variable domains were built with *SWISS-MODEL* (Waterhouse *et al.*, 2018 ▸) using the pyroglutamate-amyloid-β-specific Fab c#24 heavy chain (PDB entry 5myx, chain *B*; Piechotta *et al.*, 2017 ▸) and the VL fragment of *M. musculus* IgG from the crystal structure of the anion-exchanger domain of human erythrocyte band 3 (PDB entry 4yzf, chain *F*; Arakawa *et al.*, 2015 ▸) as the most similar templates for the VH and VL chains, respectively. These models were later aligned with the corresponding domains of the Maga_Fab_ structure (Cα r.m.s.d.s of 0.36 Å for Fab_VH_ and 15C4_VH_ and 0.38 Å for Fab_VL_ and 15C4_VL_).

**Figure 3:**
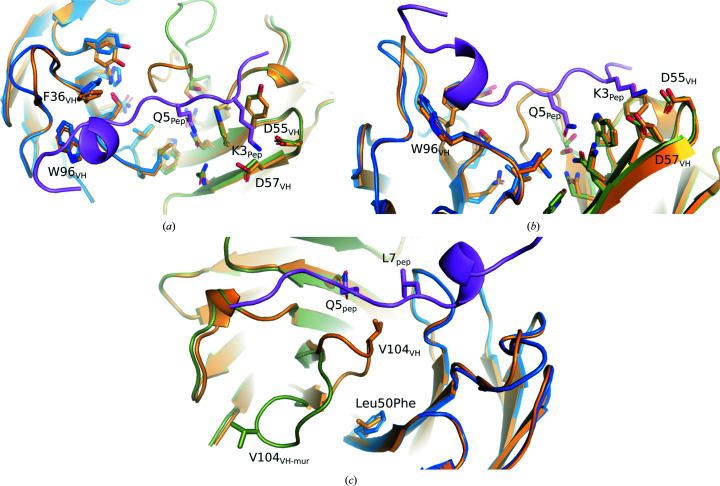
Structural alignment of murine hybridoma 15C4 homology models and the Maga_Fab_ crystal structure. (*a*) All residues interacting with the peptide and its surroundings are conserved. Maga_Fab_ is shown in orange. Murine 15C4 heavy and light variable domains are shown in green and blue, respectively. (*b*) The same as in (*a*) but rotated by 90°. (*c*) Potential flexibility of the VH loop. Murine 15C4 variable heavy domain (VH, green) shows a different conformation of the VH loop compared with the same loop in Maga_Fab_ (orange) that partially stabilizes the peptide. While the Maga_Fab_ variable light domain (VL, orange) contains a leucine at position 50, the murine homologue (blue) features a phenylalanine. These observations together could point to an important mutation location since the residue at position 50 could interact with the loop and alter its conformation, therefore modifying the affinity of Maga_Fab_ for the peptide and possibly for LRG1.

All residues involved in peptide recognition and their surroundings are conserved between Maga_Fab_ and the hybridoma 15C4 domains (Figs. 3 ▸ *a* and 3 ▸ *b*). The differences are mostly scattered across exposed areas of Maga_Fab_ that do not interact with the epitope. However, there is a difference at Leu50_Fab-VL_, a highly conserved residue amongst human immunoglobulins that is a phenylalanine in the original murine fragment. This Leu/Phe interacts with Val103_Fab-VH_, which is located at the end of a flexible loop (Arg98_Fab-VH_–Tyr107_Fab-VH_) that is involved in interaction with the peptide (Val103_VH_–Leu7_Pep_) (Fig. 3 ▸ *c*). In the 15C4 VH homology model this loop is oriented differently to in our Maga_Fab_ crystal structure but, considering that it was modeled independently of the VL chain and the peptide, one cannot assume that this conformation might be due to the Leu50Phe substitution; rather, it might be due to an orientation generated during the modeling process or the original orientation in the template used by *SWISS-MODEL*. As mentioned before, the template selected by *SWISS-MODEL* as the most similar was the pyroglutamate-amyloid-β-specific Fab c#24 heavy chain (PDB entry 5myx, chain *B*; Piechotta *et al.*, 2017 ▸), the corresponding loop of which, although three residues shorter (Arg98–Tyr104), shows the orientation predicted in the 15C4 VH homology model. Nevertheless, the Leu50Phe substitution points to a region that could be critical in stabilizing the VH loop that, at the same time, interacts with the peptide.

### 3.4. Model of the LRG1–Fab complex

In the absence of a three-dimensional structure of LRG1, analysis of its sequence and comparison with similar proteins with structures that are known can be utilized to construct a homology model of LRG1, which in turn can be used to predict the structure of its complex with Magacizumab. Analysis of the LRG1 sequence with *PSIPRED* (Buchan & Jones, 2019 ▸), *BlastP* (Altschul *et al.*, 1990 ▸), *PROSITE* (Sigrist *et al.*, 2013 ▸) and *XtalPred* (Slabinski *et al.*, 2007 ▸) suggests the presence of a 35-residue N-terminal signal peptide formed by a 17-amino-acid disordered region (Met1–Pro17) followed by a 16-residue helix (His18–Ala34). Up to eight leucine-rich repeats (LRRs) are predicted between residues 93 and 282, while the C-terminal domain is identified as a leucine-rich-repeat C-terminal domain (LRRCT).

**Figure 4:**
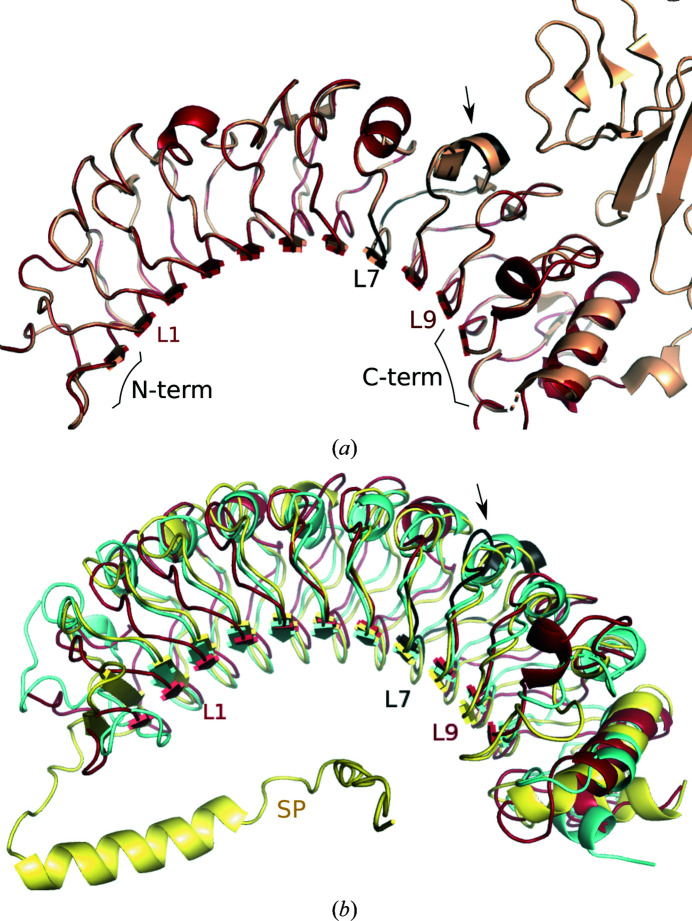
LRG1 models. (*a*) Superposition of a homology model of LRG1 (red, predicted with *SWISS-MODEL*) and NetrinG ligand 2 (NGL2; PDB entry 3zyi, chain *A*) used as the template. The arrow shows the location of the epitope recognized by Maga_Fab_ (black, LRR7). Leucine-rich repeats 1, 7 and 9 are labeled as a reference. (*b*) Superposition of the LRG1 homology model (red), the best threading model (blue) and the *ab initio* model predicted by *AlphaFold* (yellow). Leucine-rich repeats 1, 7 and 9 are labeled as a reference and the signal peptide is labeled SP.

The three-dimensional structure of LRG1 was modeled with *SWISS-MODEL* (Waterhouse *et al.*, 2018 ▸) using NetrinG ligand 2 (NGL2) as the template (PDB entry 3zyi, chain *A*; Seiradake *et al.*, 2011 ▸; 24.7% sequence identity and 32.0% sequence similarity with 86.0% coverage). Usually, it is thought that the structure of a protein can be predicted with some accuracy when its sequence identity compared with the template is greater than 30% (Xiang, 2006 ▸). For this reason, additional models will be discussed later. NGL2 is a type I transmembrane protein composed of a nine-leucine-rich-repeat domain (NGL2_LRR_), an immunoglobulin-like domain (NGL2_Ig_), a glycosylated region, a transmembrane helix and a cytoplasmic domain (Seiradake *et al.*, 2011 ▸). The resulting LRG1 model reveals a canonical leucine-rich-repeat domain (LRR domain) composed of nine leucine-rich repeats (LRRs) containing the Maga_Fab_ epitope (at LRR7), capped by N- and C-terminal domains (Fig. 4 ▸ *a*). The N-terminal domain is similar to the N-terminal cap in NGL2_LRR_ but lacks the disulfide bridge present in NGL2_LRR_ between Cys50 and Cys61 (Cys43 and Ile54 in LRG1). However, LRG1 shows a second cysteine close to Ile54 (Cys56), which could establish a disulfide bridge with Cys43 in a slightly different fold to that predicted by *SWISS-MODEL*. The LRG1 C-terminal domain was modeled very similarly to the C-terminal cap of NGL2_LRR_, containing two helices and a disulfide bridge (Cys303–Cys329) equivalent to the Cys304–Cys329 bridge in NGL2_LRR_.

Also noticeable is the fact that, as mentioned before, *BlastP* (Altschul *et al.*, 1990 ▸) and *PROSITE* (Sigrist *et al.*, 2013 ▸) sequence analyses predict only eight LRRs instead of the nine LRRs modeled by *SWISS-MODEL*. While these programs identify the first LRR beginning at residue Lys93, homology modeling predicts an additional LRR between Thr70 and Ser92, corresponding to the first LRR of NGL2_LRR_. Therefore, although the consensus LRR motif L*XX*L*X*L*XX*N/C*X*L is not found between residues 70 and 92, the abundance and location of leucine residues is similar enough to model this section as an LRR (Supplementary Fig. S5).

Sequence alignment of LRG1 and NGL2_LRR_ (excluding the NGL2_Ig_ domain) performed with *EMBOSS-Needle* resulted in an identity of 23.6% and a similarity of 43.0%. Since the identity was below 30%, additional LRG1 models were built with *RaptorX* (Peng & Xu, 2011 ▸; Xu *et al.*, 2021 ▸), a program that uses threading instead of homology as a method for structure prediction. Five additional models were predicted with *RaptorX* using the LRG1 sequence without the signal peptide as input. The estimated Cα r.m.s.d. of these LRG1 models ranged from 2.02 to 2.13 Å and they showed very similar folding of all domains compared with the homology model calculated by *SWISS-MODEL* (Fig. 4 ▸ *b*). Interestingly, although Cys43 and Cys56 do not form a disulfide bridge in these threading models, they are predicted to be close enough (7.2 Å between Cα atoms) and could potentially establish such a bond *in vivo*, while the homology model showed a slightly longer separation between these cysteines (10.0 Å between Cα atoms), preventing a bridge. The cysteines located in the C-terminal domain (Cys303–Cys329) that form a disulfide bridge in the homology model are also predicted in the same position by threading.

Finally, the recent public release of human proteome predictions by *AlphaFold* (Jumper *et al.*, 2021 ▸) allowed us to compare our LRG1 homology and threading models with a novel *ab initio* model based on the primary amino-acid sequence and deep learning. In this study, a very high per-residue confidence score (pLDDT > 90) is obtained for the entire structure, excluding the first 35 residues that correspond to the signal peptide (which were absent in our *SWISS-MODEL* homology model and the threading predictions). The folding of all domains is very similar to the homology and threading models and predicts disulfide bridges in both the N- and C-terminal domains (Fig. 4 ▸ *b*).

The similarity between the homology, threading and *ab initio* models suggests that these predictions might be relatively accurate since different methods returned comparable models. However, the crystal structure of LRG1 will be required to perform further analysis, since homology, threading and *ab initio* models generally are not sufficiently reliable for reaching deeper conclusions.

**Figure 5:**
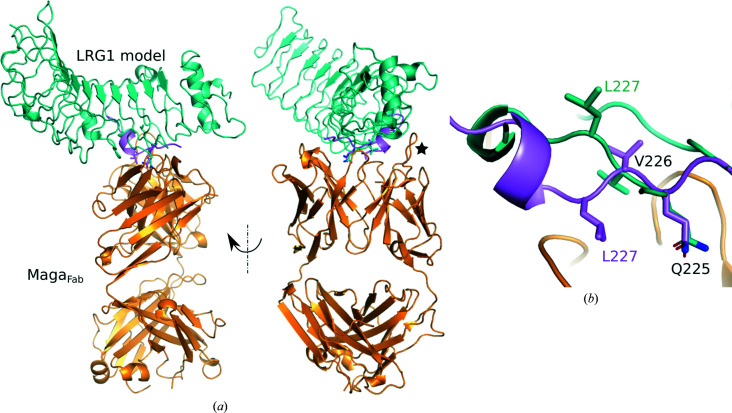
Maga_Fab_–LRG1 complex. (*a*) Superposition of the LRG1 model (green) with the crystal structure of the peptide (violet) and Maga_Fab_ (orange), taking the Cα atoms of Gln225 and Val226 as a reference, including a 90° side view. A flexible and exposed loop of Maga_Fab_ that is potentially involved in LRG1 recognition is labeled with a star. (*b*) The superposition of LRG1 LRR7 with the peptide of the crystal structure shows significant conformational differences in the main chain, probably not only due to the already described crystal contacts with the peptide but also due to inaccuracies in the LRG1 homology model.

To generate a model of whole LRG1 in complex with Maga_Fab_, we manually superposed the epitope of the *SWISS-MODEL* homology model of LRG1 with the peptide from our crystal structure (Fig. 5 ▸ *a*). The peptide crystallized in complex with Maga_Fab_ corresponds to the bold section of LRG1 LRR7 (214-LERLHLE**GNKLQVLGKDLLLPQ**PD-237); therefore, an alignment was performed based on the location of this repeat and was manually adjusted to fit the orientation of the key residue Gln225 (Gln5_Pep_) and the surrounding main chain (Fig. 5 ▸ *b*). While Gln225 and Val226 of the LRG1 homology model align well with Gln5 and Val6 of the peptide from the crystal structure, a 180° flip of the main chain (and, as a consequence, of the side chain) is observed for Leu227 (Fig. 5 ▸ *b*). In the Maga_Fab_–peptide complex crystal structure, Leu227 (Leu7_Pep_) appears to play an important role by interacting with Val103_VH_, but this interaction is not suggested by the homology model. Attempts to superpose the Cα atoms of Leu227 and Leu7_Pep_, in addition to Gln225 and Val226, resulted in clashes of LRR7 and LRR8 with a Maga_Fab_ loop (Ala25–Phe36). However, since this Maga_Fab_ loop is exposed to the solvent it is likely to be flexible, possibly allowing the binding of LRG1 without clashes and maybe even assisting in the recognition of LRR7 (Fig. 5 ▸ *a*). In addition, the crystal contacts between the peptide molecules in the asymmetric unit described above might have favored peptide conformations different from the structure of the complex in solution. Crystal structures of either LRG1 or the LRG1–Maga_Fab_ complex would allow a more complete description of the interactions leading to LRG1 recognition.

## 4. Conclusions

In this work, we have determined the three-dimensional structure of the Maga_Fab_ fragment in complex with a 15-amino-acid peptide corresponding to the epitope of LRG1. A key Gln–Gln bidentate hydrogen bond and the presence of charged residues in the surroundings comprise the main interactions between Maga_Fab_ and LRG1. This binding is assisted by secondary residues whose weaker interactions could be strengthened by the mutations suggested herein, potentially leading to an increase in the affinity of the Maga_Fab_–LRG1 complex. In addition, computational homology and threading models of LRG1 were generated and analyzed to suggest a model of the Maga_Fab_–LRG1 complex.

## 5. Related literature

The following references are cited in the supporting information for this article: Madeira *et al.* (2019 ▸) and Waterhouse *et al.* (2009 ▸).

## Supplementary Material

PDB reference: Magacizumab Fab fragment in complex with human LRG1 epitope, 7q4q

Supplementary Figures. DOI: 10.1107/S2059798322004132/gi5036sup1.pdf

## Figures and Tables

**Table 1:** X-ray diffraction data-processing and refinement statistics for the Maga_Fab_–peptide complex Values in parentheses are for the highest resolution shell.

Data collection	
Beamline	XALOC, ALBA
Detector	PILATUS 6M
Space group	*P*2_1_2_1_2_1_
*a*, *b*, *c* (Å)	64.5, 76.9, 217.7
α, β, γ (°)	90, 90, 90
Wavelength (Å)	0.979
Scaling	
Resolution (Å)	49.5–1.65 (1.68–1.65)
*R*_merge_ (%)	7.5 (178.6)
*R*_p.i.m._ † (%)	2.1 (50.0)
CC_1/2_	0.999 (0.632)
〈*I*/σ(*I*)〉	18.3 (1.6)
Completeness (%)	100 (100)
Multiplicity	13.1 (13.5)
Refinement	
Resolution (Å)	49.5–1.65
No. of reflections	131170
*R*_work_/*R*_free_ ‡ (%)	18.3/20.7
No. of atoms	
Protein	7125
Peptide	230
Mean *B* factor (Å^2^)	35.1
R.m.s.d.	
Bond lengths (Å)	0.006
Angles (°)	0.902

† Tickle *et al.* (2000 ▸).

‡ Evans (2011 ▸).

**Table 2:** Interface surface areas due to crystal contacts Interface surface areas between the Maga_Fab_ chains (VL and VH), the peptide (Pep) and symmetry-related chains (marked with an asterisk) calculated with the *PISA* server (Krissinel & Henrick, 2007 ▸). The interface surface areas between the main peptide (Pep) and main Maga_Fab_ chains (VL and VH) are included as a reference. Numerous residues are involved in these contacts, mainly through hydrogen-bond and hydrophobic interactions.

Chain 1	Chain 2	Interface surface area (Å^2^)
VL* (chain *C*)	VL (chain *A*)	262.5
	VH (chain *B*)	0
	Pep (chain *E*)	14.2
VH* (chain *D*)	VL (chain *A*)	31.6
	VH (chain *B*)	0
	Pep (chain *E*)	187.1
Pep* (chain *F*)	VL (chain *A*)	78.0/71.4†
	VH (chain *B*)	210.2
	Pep (chain *E*)	352.0
Pep (chain *E*)	VL (chain *A*)	338.2
	VH (chain *B*)	356.0

† The second value is an interaction with a crystal contact VL (Asn2_Pep*_–Ser207_VLneigh_).
