# Supplementary material for: Structural basis of human LRG1 recognition by Magacizumab, a humanized monoclonal antibody with therapeutic potential
Source: Acta Crystallogr D Struct Biol. 2022 May 9;78(Pt 6):725–34. doi: 10.1107/S2059798322004132 (PMC9159282; doi:10.1107/S2059798322004132)
Supplement: Supplementary file 1 [file d-78-00725-sup1.pdf]

STRUCTURAL BIOLOGY

Volume 78 (2022)

Supporting information for article:

Structural basis of human LRG1 recognition by Magacizumab, a humanized monoclonal antibody with therapeutic potential

Javier Gutiérrez-Fernández, Faiza Javaid, Giulia De Rossi, Vijay Chudasama, John Greenwood, Stephen E. Moss and Hartmut Luecke

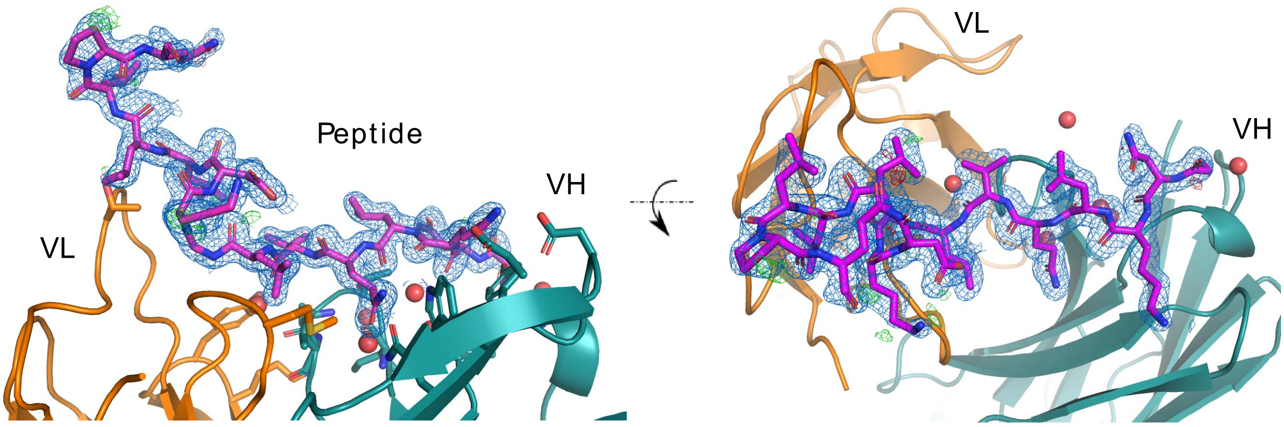

Figure S1 Electron density of LRG1 epitope peptide. Electron density maps of the LRG1 epitope peptide bound to $Maga_{Fab}$. $2F_o$-F_c density map, contoured at 1σ, is shown in blue. The F_o-F_c density map, contoured at 3σ, is shown in green and red for positive and negative density respectively.

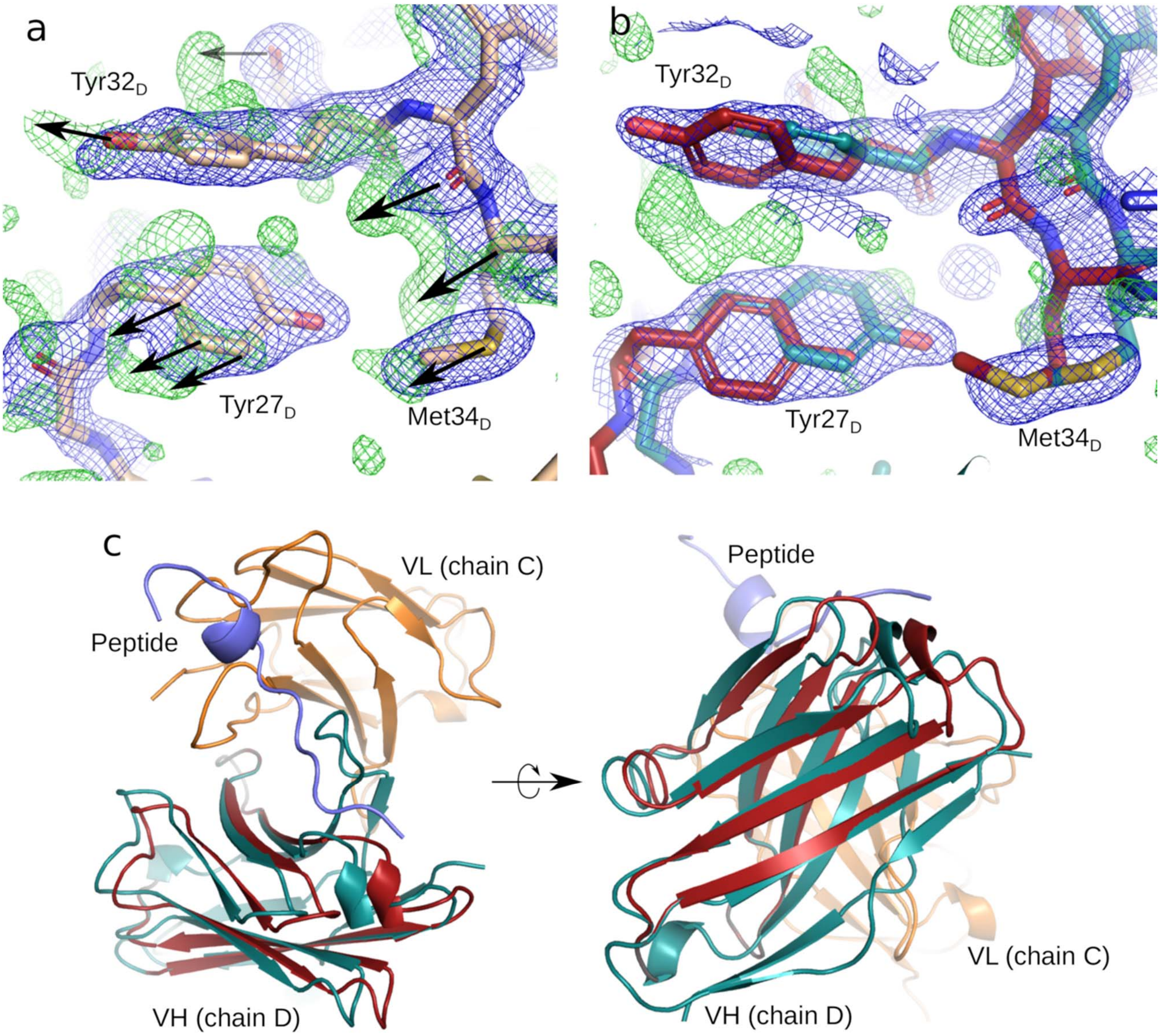

Figure S2 Maga$_{Fab}$ VH double conformation. **a)** Electron density maps of a section of Maga$_{Fab}$ VH built as a single conformation. $2F_o$-F_c density map, contoured at 1σ, is shown in blue. The F_o-F_c density map, contoured at 3σ, is shown in green. A significant amount of positive density in the F_o-F_c map after refinement suggests a possible translation of a fraction of the entire region in the direction of the arrows. **b)** Electron density maps of the same section as **a**, built as a double conformation (conformation with occupancy of 0.47 shown in red, conformation with occupancy 0.53 shown in green) and refined with the same strategy. $2F_o$-F_c and F_o-F_c maps shown with same colors and contours as in **a**. **c)** Overall structure of the variable chains of the Maga$_{Fab}$ molecule 2, showing both conformations for the VH.

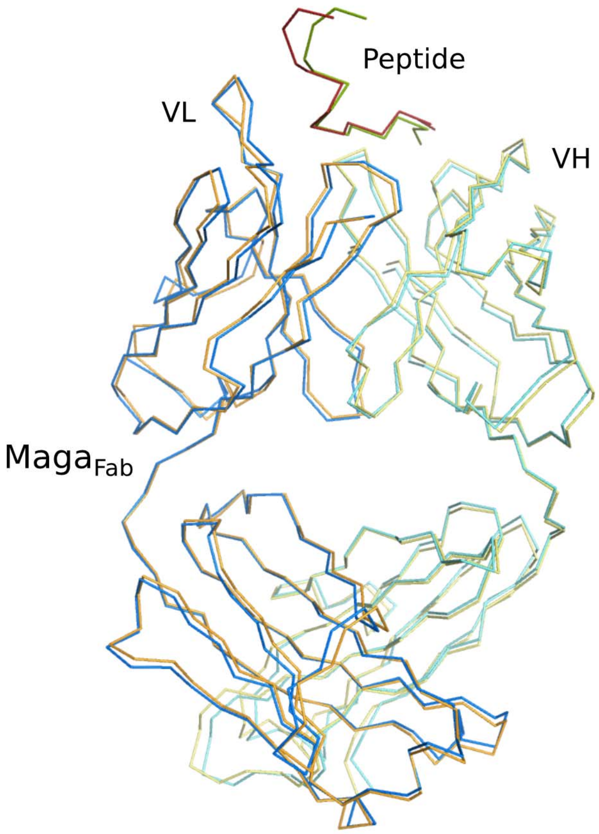

Figure S3 Maga$_{Fab}$ in complex with LRG1 epitope peptide. General representation of both Maga$_{Fab}$ molecules present in the asymmetric unit of the crystal, in complex with LRG1 epitope peptide. Variable light (VL) and variable heavy (VH) chains orange and yellow correspond to one Maga$_{Fab}$ molecule, binding the peptide shown in red. Blue and cyan VL and VH chains correspond to the second Maga$_{Fab}$ molecule binding the peptide colored in green.

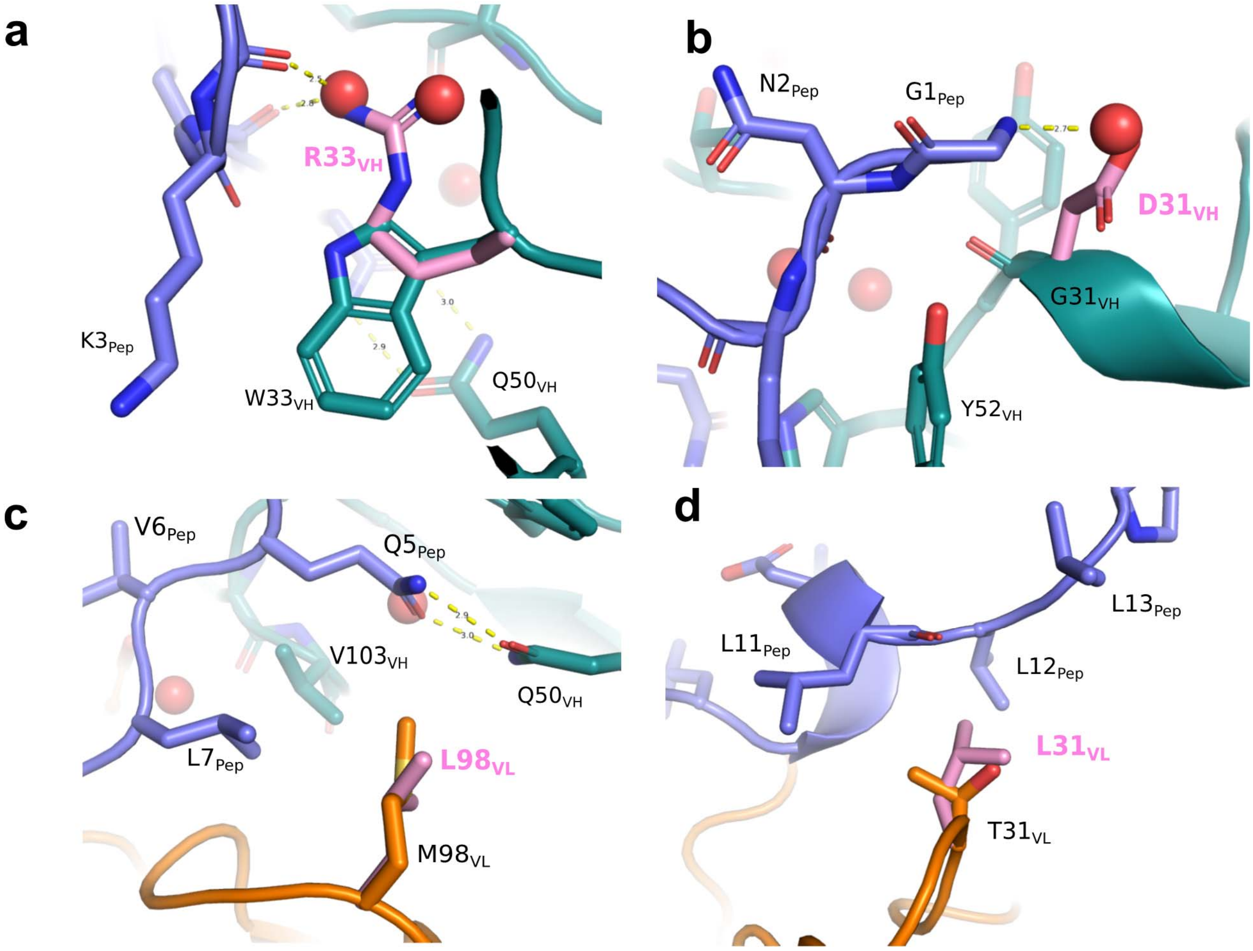

Figure S4 Potential mutations to increase the affinity of Magacizumab for LRG1. Suggested point mutations that could increase the affinity of Magacizumab for LRG1, colored and labeled in pink and superimposed over the original amino acid. **a)** W33$_{VH}$R. **b)** G31$_{VH}$D. **c)** M98$_{VL}$L. **d)** T31$_{VL}$L. Their orientations are based on their most probable rotamer conformation, with the exception of W33R (a), which was modeled fulfilling all geometric parameters to fit into the electron density of key water molecules.

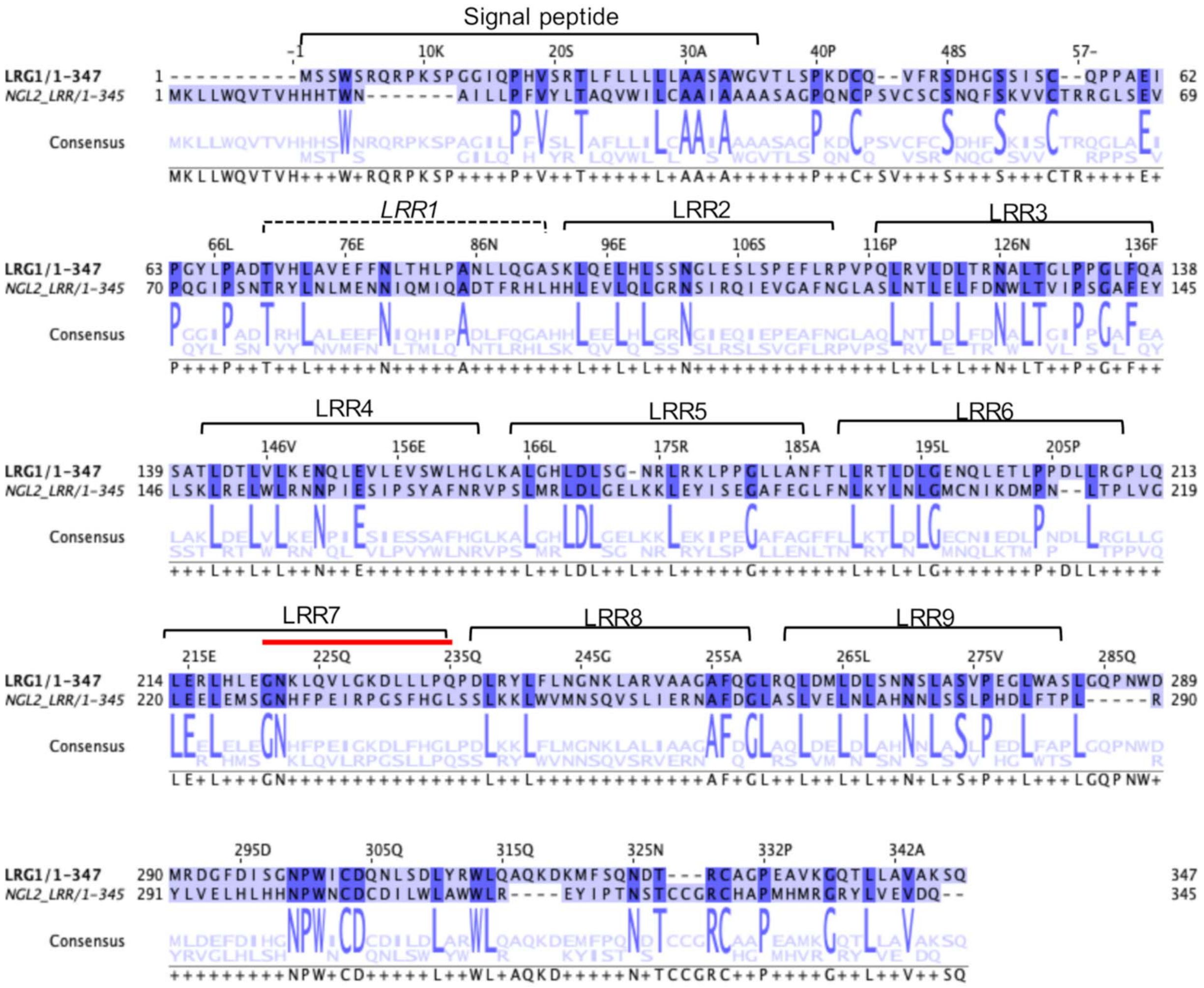

Figure S5 LRG1-NGL2$_{LRR}$ sequence alignment and LRR domains. Pairwise sequence alignment between LRG1 and NGL2, performed by EMBOSS Needle (Madeira *et al.*, 2019) and represented in Jalview (Waterhouse *et al.*, 2009). Residue numbering corresponds to LRG1 protein, colors are based on identity. LRRs from 2 to 9 are shown as predicted by Prosite (Sigrist *et al.*, 2013). LRR1 is not predicted from the sequence by any of the programs used in this work, although the corresponding region (70-92) is modeled as such by SWISS-MODEL. The red line indicates the epitope recognized by Maga$_{Fab}$.
